# Chilling-Mediated DNA Methylation Changes during Dormancy and Its Release Reveal the Importance of Epigenetic Regulation during Winter Dormancy in Apple (*Malus* x *domestica* Borkh.)

**DOI:** 10.1371/journal.pone.0149934

**Published:** 2016-02-22

**Authors:** Gulshan Kumar, Usha Kumari Rattan, Anil Kumar Singh

**Affiliations:** 1 Department of Biotechnology, CSIR-Institute of Himalayan Bioresource Technology, Palampur 176061, HP, India; 2 Academy of Scientific and Innovative Research, New Delhi, India; National Institute of Plant Genome Research (NIPGR), INDIA

## Abstract

Winter dormancy is a well known mechanism adopted by temperate plants, to mitigate the chilling temperature of winters. However, acquisition of sufficient chilling during winter dormancy ensures the normal phenological traits in subsequent growing period. Thus, low temperature appears to play crucial roles in growth and development of temperate plants. Apple, being an important temperate fruit crop, also requires sufficient chilling to release winter dormancy and normal phenological traits, which are often associated with yield and quality of fruits. DNA cytosine methylation is one of the important epigenetic modifications which remarkably affect the gene expression during various developmental and adaptive processes. In present study, methylation sensitive amplified polymorphism was employed to assess the changes in cytosine methylation during dormancy, active growth and fruit set in apple, under differential chilling conditions. Under high chill conditions, total methylation was decreased from 27.2% in dormant bud to 21.0% in fruit set stage, while no significant reduction was found under low chill conditions. Moreover, the demethylation was found to be decreased, while methylation increased from dormant bud to fruit set stage under low chill as compared to high chill conditions. In addition, RNA-Seq analysis showed high expression of DNA methyltransferases and histone methyltransferases during dormancy and fruit set, and low expression of DNA glcosylases during active growth under low chill conditions, which was in accordance with changes in methylation patterns. The RNA-Seq data of 47 genes associated with MSAP fragments involved in cellular metabolism, stress response, antioxidant system and transcriptional regulation showed correlation between methylation and their expression. Similarly, bisulfite sequencing and qRT-PCR analysis of selected genes also showed correlation between gene body methylation and gene expression. Moreover, significant association between chilling and methylation changes was observed, which suggested that chilling acquisition during dormancy in apple is likely to affect the epigenetic regulation through DNA methylation.

## Introduction

The epigenetic modifications including DNA methylation and histone methylation/acetylation, are the heritable information [1], which modulate the gene expression without changing the DNA sequence. Therefore, these modifications provide an additional regulatory mechanism to influence the genes expression in response to internal or external environmental factors. Epigenetic regulation involves the post-translational histone protein modifications and DNA methylation [2, 3]. Epigenetic mechanisms have been known to modulate plant growth and development to adapt under environmental stresses. The methylation at cytosine base is well known strategy employed by plants to adopt the new environmental conditions without altering its DNA sequence [4]. The role of cytosine based DNA methylation in epigenetic regulation has been established and extensively studied in various plant species. Change in methylation pattern and chromatin modification of genomic DNA has been reported in various developmental processes and environmental stresses in plants [5]. These changes in methylation pattern have also been associated with transcriptional regulation in various plants [6–9]. The cytosine methylation is mediated by cytosine methyltransferases and is inheritable across the generations, while the cytosine demethylation is actively catalyzed by DNA glycosylases, beside the passive removal by replication process [10]. The cytosine methylation in higher plants occur predominantly at CG or CHG sequences (where H represents A, C, or T) [11–14] and sometimes at CHH sequences [15]. Irrespective of these sequence contexts, DNA cytosine methyltransferase, DOMAINS REARRANGED METHYLTRANSFERASE 2 (DRM2) catalyzes the *de novo* cytosine DNA methylation in plants. While, DNA METHYLTRANSFERASE 1 (MET1) maintains CG methylation; plant-specific DNA methyltransferase, CHROMOMETHYLASE 3 (CMT3) maintains CHG methylation and DRM2 maintains CHH methylation [10]. Whereas, the active removal of cytosine methylation is catalyzed by DEMETER (DME) and REPRESSOR OF SILENCING 1 (ROS1), and their paralogs DEMETER-LIKE 2 (DML2) and DEMETER-LIKE 3 (DML3), which are the members of DNA glycosylase family [10, 16, 17].

Although, DNA methylation occurs mainly in the transposable elements, methylation within the promoter and transcribed regions of the genes is common and correlated with their transcriptional activity [18–21]. Thus, it is apparent that cytosine methylation regulates various developmental and adaptive processes. Among all the presently available techniques to determine the cytosine methylation in genomic DNA, Methylation sensitive amplified polymorphism (MSAP) is reliable, cost effective and most widely used technique [22]. MSAP is a variant of amplified fragment length polymorphism technique (AFLP), and is widely used to study the changes in methylation pattern of genomic DNA. MSAP profile is based on the differential sensitivity of two isoschizomers, *Hpa*II and *Msp*I to methylation of their recognition sequences (CCGG). Activity of both the enzymes, *Hpa*II and *Msp*I is affected by the methylation state of external and internal cytosine residues. The full methylation (both strand methylated) of cytosine makes the *Hpa*II inactive to cleave, but hemi-methylation (single strand methylated) site has no effect on its activity. Whereas *Msp*I cleaves hemi or fully methylated site when internal cytosine is methylated (C^5m^CGG), while methylation of external cytosine (^5m^CCGG) inhibits activity of *Msp*I [23].

Apple (*Malus* x *domestica* Borkh.) is an important fruit tree and cash crop for most of the temperate regions of the world. The low temperature of winter season plays crucial role in phenological events of apple tree. As winter approaches, the plant undergoes dormancy in order to survive the chilling temperature of winters. After accumulation of sufficient chilling, the dormant buds resume active growth in response to warm temperature of spring season. This phenomenon of bud dormancy during winter season is well understood and divided into three phases known as paradormancy, endodormancy and ecodormancy [24, 25]. It is reported that changing climatic conditions with high winter temperature resulted in improper flower development in tree species with high chilling requirement for winter dormancy release, which affect the productivity and quality of the developing fruit [3, 26]. Thus, chilling temperature during winter dormancy appears to have remarkable effect on quality and productivity of fruits. However, the molecular mechanism of chilling mediated dormancy release is still obscure and is a matter of debate. The understanding of chilling mediated dormancy release at genetic level may have profound impact on development of climate resilient cultivars.

The low temperature associated changes in DNA methylation have been evident from the several previous studies [11, 27–30]. However, no study related to changes in DNA methylation during dormancy period and its release in apple has been performed, so far. Therefore, to study the cytosine methylation based epigenetic regulation of chilling mediated dormancy release in apple, MSAP approach was employed to analyze the changes in cytosine methylation pattern during dormancy break and subsequent fruit set. In order to elucidate the effect of chilling temperature on methylation, the samples of Royal delicious apple variety were collected from two different locations in Kullu valley of Himachal Pradesh, India, with more than 1000 m altitude difference. A recent study by Jangra and Sharma [31] reported rise in average temperature during winter season in low altitude regions of Kullu valley. Therefore, the selected location with low altitude was assumed to receive less chilling during winter seasons as compared to location at high altitude. The Royal delicious is a high chill variety with >1200 chilling units (one chilling unit is equal to temperature below 7°C for one hour) requirement during dormancy period to resume normal active growth. In addition, the expression level of differentially methylated genes as well as genes involved in *de novo* cytosine methylation, its maintenance and active removal of cytosine methylation was analyzed in various stages of bud dormancy, dormancy release and fruit set using RNA-seq data of apple generated through Illumina Genome Analyzer IIx.

## Materials and Methods

### Plant material

For sample collection, apple trees of Royal delicious cultivar were selected from two private apple orchards situated at Palchan (32° 18′ 36″ N, 77° 10′ 40 E″; altitude 2350 m) and Seobag (31° 58′ 57″ N, 77° 07′ 43 E″; altitude 1250 m) of Kullu district, Himachal Pradesh (HP), India with owner’s permission. The selected locations have differential chilling availability during the winter season, where Seobag has less chilling availability, while the Palchan has more chilling availability during winter season. The daily maximum and minimum temperature data was obtained from Hill Agricultural Research & Extension Centre, Kullu (HP) and DRDO-Snow and Avalanche Study Estt. (SASE) station Bahang, Manali (HP) for Seobag and Palchan sampling locations, respectively. The samples comprising of four developmental stages, namely dormant bud (DB), silver tip (ST), green tip (GT) and initial fruit set (FS) were collected during the January 8, 2013 to April 15, 2013 from Seobag and during January 8, 2013 to May 8, 2013 from Palchan (Table 1). Each sample was collected from three trees and immediately frozen in liquid nitrogen and stored at – 80°C for further use.

**Table 1:** The sample abbreviation and their collection intervals.

Developmental stages	Tissue type	Low chill location (Seobag)		High chill location (Palchan)	
		Sample abbreviation	Collection intervals	Sample abbreviation	Collection intervals
Bud	Dormant bud	DBL	0 day	DBH	0 day
	Silver tip	STL	45 day	STH	56 day
Bud break	Green tip	GTL	57 day	GTH	73 day
Fruit	Initial fruit set	FSL	96 day	FSH	119 day

### DNA extraction

The DNA was extracted as describe by Xin and Chen [32], with minor modifications. Briefly, the frozen tissue was crushed to powder in liquid nitrogen with activated charcoal and DNA extraction buffer (100mM Tris, pH 8.0; 20mM EDTA; 2% CTAB; 1.4M NaCl; freshly added 0.2% β-mercaptoethanol) was added and incubated at 60°C for 1 hr. After incubation, chloroform:isoamyl alcohol (24:1) treatment was given and 2 volumes of dilution buffer (100mM Tris, pH 8.0; 20mM EDTA; 2% CTAB) was added to the aqueous phase. The diluted samples were incubated at 60°C until DNA-CTAB precipitate starts appearing. The precipitate was washed with 70% ethanol and re-suspended in high salt TE buffer supplemented with RNase A (10mM Tris, pH 8.0; 2mM EDTA; 1M NaCl; 50μg/ml RNase), and further incubated at 60°C for 30 min. The RNase treated DNA was ethanol precipitated and re-suspended in TE buffer. The DNA was treated with proteinase K to remove any protein impurities which may interfere with restriction digestion of genomic DNA.

### Methylation sensitive amplified polymorphism assay

The MSAP protocol was followed as described by Portis et al. [33] with minor modification. Briefly, 500 ng genomic DNA was digested with *Eco*RI and divided in to two equal halves for digestion with *Hpa*II and *Msp*I. The double digested DNA was subjected to proteinase K treatment and purified through ethanol precipitation. The quantification of restriction digested DNA was done using fluorescence based broad range Qubit assay (Invitrogen, USA). The 100 ng DNA of each digestion reaction was ligated to *Eco*RI and *Hpa*II/*Msp*I specific adaptors (S1 Table). The adaptor ligated DNA was 1/10 diluted and used as a template in pre-amplification PCR. The 1/20 diluted pre-amplification product was used as template for selective amplification PCR using 16 combinations of *Hpa*II/*Msp*I and *Eco*RI adaptor specific primers (S1 Table). Selectively amplified PCR products were mixed with equal amount of formamide dye (98% formamide, 10mM EDTA, 0.01% bromophenol blue w/v and 0.01% w/v xylene cyanol) and denatured at 95°C for 5 min. The denatured samples were separated on 6% denaturing sequencing gel and silver stained to visualize the DNA fragments.

### Scoring and analysis of methylation patterns

The bands appeared on sequencing gel were scored according to Karan et al. [34]. Briefly, four types of bands were scored: type I fragments represent unmethylated and were present in both the *Eco*RI/*Msp*I and *Eco*RI/*Hpa*II lanes; type II fragments represent hemi-methylated loci and were present only in *Eco*RI/*Hpa*II lane; type III fragments represent fully methylated loci and were present only in *Eco*RI/*Msp*I lane; the type IV fragments represent the methylated/demethylated loci in two different samples which is indicated by absence of band in either enzyme combination in one of the samples.

### Sequencing and analysis of polymorphic fragments

The randomly selected polymorphic fragments were excised from the gel, crushed in 20μl TE buffer and incubated at 94°C for five min. The eluted fragments were re-amplified with the same primer set and PCR thermal conditions as used for the corresponding selective amplification. The PCR products were cloned in pTZ57R/T TA cloning vector (Thermo Fisher Scientific). The plasmids were isolated from positive recombinant clones and sequenced using Big Dye Terminator v3.1 (Applied Biosystems, USA) with M13 forward and reverse primers. The resulted sequences were searched against phytozome (www.phytozome.net) as well as NCBI non-redundant database using BLASTN to find the genes and their flanking regions (within 1Kb) associated with methylation as identified through MSAP.

### RNA sequencing and data analysis

Total RNA was extracted from 100 mg of plant material using iRIS solution [35], and quantified using NanoDrop UV-VIS spectrophotometer. The integrity of RNA was further checked on denaturing formaldehyde agarose gel. Eight RNA-Seq libraries, including DBL, STL, GTL, FSL and DBH, STH, GTH, FSH stages under low and high chill conditions, respectively, were prepared, using TruSeq RNA sample preparation kit v2 as per manufacturer’s instructions (Illumina Inc., USA). The quantification and insert size validation of libraries was done using Qubit dsDNA BR assay kit (Life technologies, USA) and Agilent Bioanalyzer DNA 1000 chip (Agilent Technologies), respectively. The paired-end (2×76) sequencing of RNA-Seq libraries was performed on Genome Analyzer IIx (Illumina) after cluster generation using cluster station (Illumina Inc., USA). The generated reads were *de novo* assembled on CLC Genomics Workbench 6.5 (http://www.clcbio.com) after quality filtering using NGS QC toolkit [36]. The assembled contig file was used as database to identify the MSAP loci associated transcripts using BLASTN with e-value cutoff 1e-05. The raw reads obtained from Illumina GAIIx of all the analyzed samples were submitted as BioProject (PRJNA306594) to NCBI Sequence Read Archive under accession number SRP067619.

### Bisulfite sequencing

Two microgram of high quality genomic DNA from each of the eight samples corresponding to four developmental stages under differential chilling conditions was used for bisulfite treatment using Epitect Bisulfite Kit (Qiagen, USA) following the manufacturer’s instructions. The bisulfite converted DNA samples were used to amplify the four randomly selected polymorphic MSAP fragments; MDC013945.282, MDC019410.118, MDC011209.233 and MDC044052.10. Briefly, 2μl of bisulfite converted genomic DNA was used as template in 25μl of PCR reaction using hot start Advantage 2 polymerase mix (Clontech, USA) with touch-down thermal PCR profile. The primers corresponding to bisulfite converted polymorphic loci were designed using Methprimer (S2 Table), an online tool for designing bisulfite-conversion based Methylation PCR Primers [37]. The PCR amplified products were gel eluted using QIAEX II gel extraction kit (Qiagen, USA) and cloned in pTZ57R/T TA cloning vector. The transformants were confirmed by colony PCR and further sequencing of plasmid was performed using Big Dye Terminator v3.1 (Applied Biosystem, USA) with M13 forward and reverse primers. The sequence of unconverted DNA was used to compare the methylation status of selected polymorphic loci, where change from C (cytosine) in unconverted DNA to T (thymine) in bisulfite converted DNA represents non-methylated C, while no change from C to T represents the methylated C. The percentage of methylated cytosines was calculated using formula: [number of methylated cytosine/total number of cytosine present in different contexts] x 100.

### Quantitative real-time PCR (qRT-PCR) analysis

For qRT-PCR analysis, 1μg of total RNA was converted into first strand cDNA using RevertAid RNAse H minus cDNA synthesis kit as per manufacturer’s instructions (Fermentas Life Sciences, USA). The primers (S3 Table) were designed using Primer Express software version 3.0.1 (Invitrogen). The 10 randomly selected genes associated with polymorphic fragments were analyzed for their expression levels using qRT-PCR based method as described earlier by Arya et al. [38]. The qRT-PCR data was normalized using GAPDH gene as internal control [39]. The relative expression of candidate target genes was calculated using REST software (Qiagen) considering high chill conditions as control.

## Results

### Methylation profiling of apple genomic DNA during chilling acquisition and fruit set

**Fig 1:**
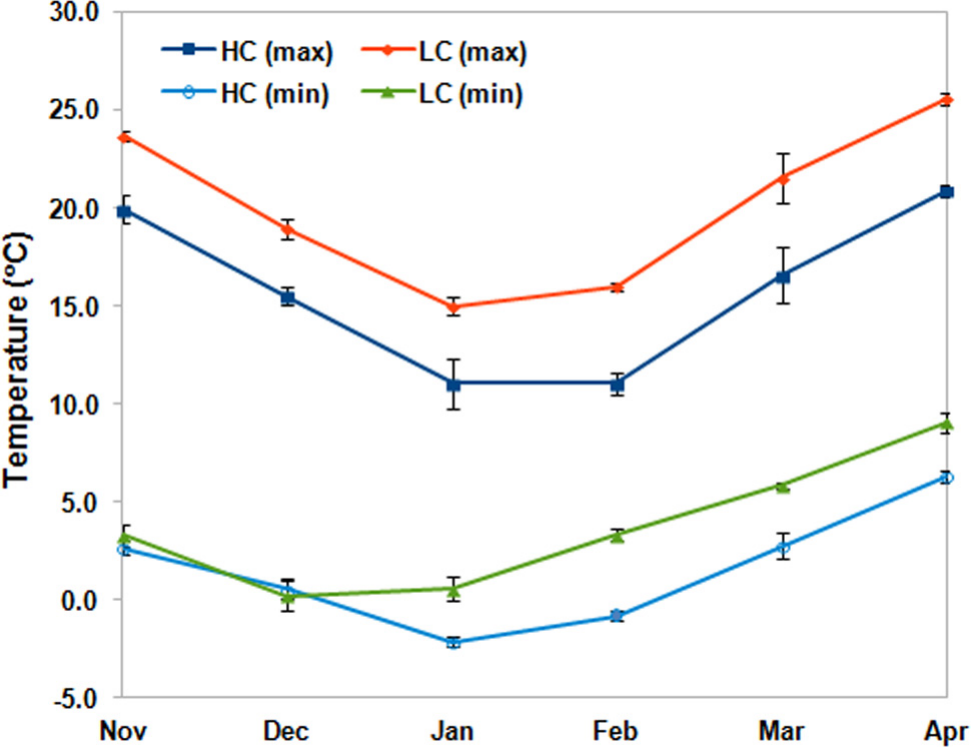
The graph showing the average of minimum and maximum daily temperature during November to April for three consecutive years (2011–2014) at both the sampling locations. The HC and LC represent the high chill condition (Palchan) and low chill condition (Seobag), respectively. The min. and max. represent the minimum and maximum temperature, respectively.

**Fig 2:**
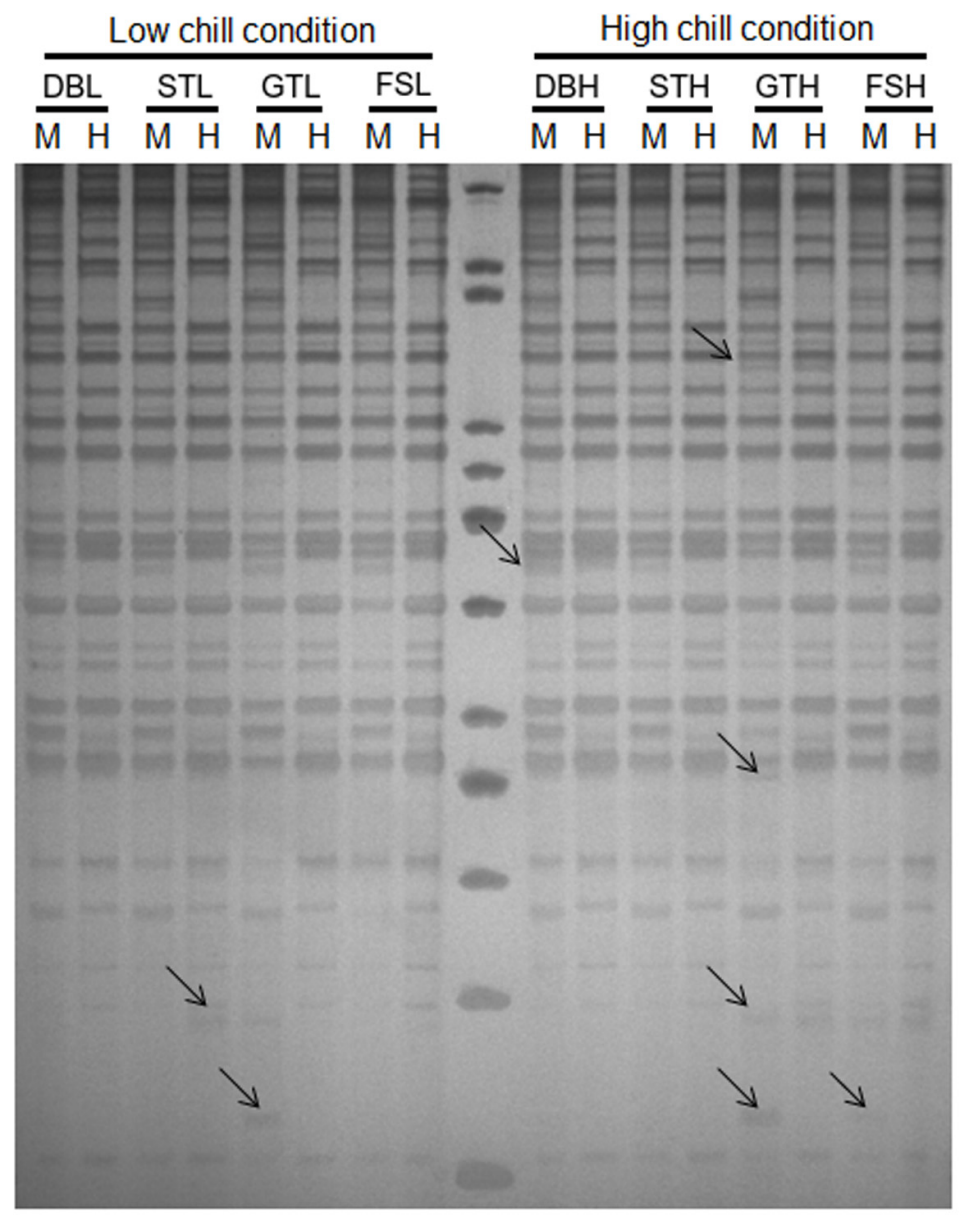
A representative gel image of MSAP assay showing polymorphic fragments in different samples. lane H: DNA digested with *Eco*RI–*Hpa*II; lane M: DNA digested with *Eco*RI–*Msp*I. The four developmental stages *viz*. Dormant bud (DB), Silver tip (ST), Green tip (GT) and Initial fruit set (FS) in apple, under low (L) chill and high (H) chill conditions, were used for MSAP analysis. The arrow marks show presence of polymorphic fragments.

The MSAP profiling of apple genomic DNA in four developmental stages *viz*, dormant bud (DB), silver tip (ST), green tip (GT) and initial fruit set (FS) under differential chilling conditions was performed using 16 primer combinations. The temperature map for both the sampling locations showed difference in minimum and maximum temperature during the winters and onset of spring season (Fig 1). The scoring of fragments (ranging between 100bp to 1Kb) resulted in identification of 2306 fragments in four stages under low chill conditions, while, 2414 fragments were observed under high chill conditions (Fig 2, Table 2). Under high chill conditions, 1810 non-methylated fragments present in both *Eco*RI/*Hpa*II and *Eco*RI/*Msp*I lanes (type I), were identified, while under low chill conditions, 1740 non-methylated fragments were identified. Under low chill conditions, higher number (166) of hemi-methylated fragments, present only in *Eco*RI/*Hpa*II lane (type II) were identified as compared to high chill conditions (132). Whereas, fully methylated fragments, present only in *Eco*RI/*Msp*I lane (type III) were found to be almost equal under low chill (232) and high chill (234) conditions. The fragments, absent in both, *Eco*RI/*Msp*I and *Eco*RI/*Hpa*II lanes (type IV), were found to be more under high chill conditions (238) as compared to low chill conditions (168) (Table 2). The total methylation percentage was found to be 24.5% and 25% under low and high chill conditions, respectively. However, percentage of the fully methylated fragments (type III and IV) was slightly higher (19.6%) under high chill conditions than under low chill conditions (17.3%). In contrast, percentage of hemi-methylated fragments (type II bands) was found to be more (7.2%) under low chill conditions than under high chill conditions (5.5%). However, the percentage of fully methylated fragments was found to be gradually decreased along with the dormancy break and fruit set under high chill conditions. The chi-square test of independence between chilling availability during dormancy period and methylation patterns suggested association between chilling and methylation level (Table 2).

**Table 2:** Changes in DNA methylation under high and low chill conditions during various stages of dormancy, dormancy release and fruit set.

MSAP band type		Dormant bud		Silver tip		Green tip		Initial fruit set		Total methylation	
		DBH	DBL	STH	STL	GTH	GTL	FSH	FSL	High chill condition	Low chill condition
Fragments type I		444	458	446	438	466	434	454	410	1810	1740
Fragments type II		33	32	37	50	30	35	32	49	132	166
Fragments type III		50	50	59	54	62	76	63	52	234	232
Fragments type IV		83	62	70	43	59	34	26	29	238	168
Total fragments		610	602	612	585	617	579	575	540	2414	2306
Chi-square test statistics	χ2	3.22		**8.08**		**8.46**		5.93		**14.87**	
	P-value	0.358		**0.044**		**0.03**		0.115		**0.002**	
**Methylation percentage**											
Total methylation (%)		27.2	23.9	27.1	25.1	24.5	25.0	21.0	24.1	25.0	24.5
Fully methylated fragments (%)		21.8	18.6	21.1	16.6	19.6	19.0	15.5	15.0	19.6	17.3
Hemi-methylated fragments (%)		5.4	5.3	6.0	8.5	4.9	6.0	5.6	9.1	5.5	7.2

### Dynamics of methylation/demethylation events in relation to chilling availability

The apple cultivar Royal Delicious, taken for study is a high chill variety and requires high chilling (>1200 CU) during dormancy period for subsequent quality fruit development. Therefore, high chill condition was considered as control to study the methylation/demethylation events in four different comparative conditions, including DBL-vs-DBH, STL-vs-STH, GTL-vs-GTH and FSL-vs-FSH during dormancy break to fruit set. The comparative MSAP profiling under differential chilling conditions showed changes in methylation and demethylation events. The differential banding patterns were considered to analyze the methylation and demethylation status of 5'-CCGG-3' sites in apple genome (Table 3). The comparative analysis revealed that methylation events under low chill conditions were increased from 0.3% in DBL-vs-DBH to 3.3% in FSL-vs-FSH, while the demethylation events were decreased from 6.5% in DBL-vs-DBH to 2.8% in FSL-vs-FSH (Table 3). The change in methylation and demethylation events in DBL-vs-DBH to GTL-vs-GTH and DBL-vs-DBH to FSL-vs-FSH comparisons were also found to be statistically significant with chi-square test of independence (S4 Table). These changes in methylation and demethylation events suggested that availability of high chilling during dormancy period was associated with progressive demethylation events during dormancy break and fruit set.

**Table 3:** DNA methylation under low chill conditions with respect to high chill conditions in various developmental stages during dormancy, dormancy release and fruit set.

Band Pattern type	*Class of banding pattern	Banding pattern				Developmental stages			
		Control		Treatment					
No change		*MspI*	*HpaII*	*MspI*	*HpaII*	DBL-vs-DBH	STL-vs-STH	GTL-vs-GTH	FSL-vs-FSH
	A	1	1	1	1	432	420	420	402
	B	0	1	0	1	23	30	28	27
	C	1	0	1	0	44	50	56	49
	D	0	0	0	0	62	43	34	29
	Total					561	543	538	507
	Percentage					93.2	92.8	92.9	93.9
**Demethylation**									
	E	0	1	1	1	10	2	0	0
	F	1	0	1	1	8	6	8	2
	G	0	0	1	1	8	10	6	6
	H	1	0	0	1	0	2	0	1
	I	0	0	0	1	7	11	5	4
	J	0	0	1	0	6	3	8	2
	Total					39	34	27	15
	Percentage					6.5	5.8	4.7	2.8
**Methylation**									
	K	1	1	0	1	2	7	2	17
	L	1	1	1	0	0	1	12	1
	M	0	1	1	0	0	0	0	0
	N	1	1	0	0	0	0	0	0
	O	0	1	0	0	0	0	0	0
	P	1	0	0	0	0	0	0	0
	Total					2	8	14	18
	Percentage					0.3	1.4	2.4	3.3

*Alphabets A-P represent classes of banding patterns.

### Analysis of MSAP polymorphic fragments

The polymorphic fragments representing methylation/demethylation events in different samples during DB to FS under differential chilling conditions were randomly selected and sequenced. The size of polymorphic DNA fragments ranged between 62 to 307 bp. From the total sequenced fragments, 47 fragments were found to be either within the gene body or within the 1Kb upstream/downstream regions of protein coding genes (Table 4). The BLASTN analysis of sequenced fragments showed that these were related to AUX/IAA27 B, pentatricopeptide repeat domain containing protein, NAC domain containing protein, DTT domain containing protein (Homeodomain-like), molecular chaperone (dnaJ), histone acetyltransferase HAC1-like, exonuclease, leucine-rich repeat receptor-like protein kinase, cytochrome P450, pectinesterase 3 (involved in cell wall modification), solute carrier family protein, ribosomal protein kinase G11A-like, acid phosphatase1-like, thioredoxin-like protein, cation efflux protein, serine/threonine-protein phosphatase, SNARE protein, tRNA-nucleotidyltransferase (ACT domain-containing protein), sodium/hydrogen exchanger family, NADH-cytochrome B5 reductase, etc. (Table 4). Most of these genes were related to cellular metabolism, DNA/RNA processing, stress response, antioxidant system and transcriptional regulation. Therefore, annotation of polymorphic fragments suggests that methylation might regulate the wide range of molecular and cellular functions during dormancy release and fruit set in apple.

**Table 4:** List of selected MSAP polymorphic fragments, their annotation, their location in apple genome and their corresponding contigs identified in RNA-Seq data.

Sl. No	Methylation status	Tissue type	Gene annotation	E-value	Associated apple protein	Fragment position	MDC ID	RNA-seq contig
1	Demethylated	DB	PREDICTED: wall-associated receptor kinase 2-like	1.4E-39	MDP0000216164	exon	MDC022332.50	C_47891
2	Demethylated	DB	DNA-directed RNA polymerases II and IV subunit 5A	5E-30	MDP0000305507	100bp 3'UTR	MDC017731.272	C_10585
3	Demthylated	DB	NAC domain-containing protein 90-like	6E-79	MDP0000130797	exon	MDC018994.343	C_56817
4	Demthylated	DB	Homeodomain-like transcriptional regulator isoform 2	1.1E-87	MDP0000194500	exon and 3'UTR	MDC009126.325	C_38969
5	Methylated	DB	Hydroxymethylglutaryl-CoA lyase	8.3E-70	MDP0000168020	700 bp 3'UTR	MDC004192.624	C_15455
6	Methylated	DB	probable alpha,alpha-trehalose-phosphate synthase	3.3E-62	MDP0000257194	intron	MDC004525.265	C_12925
7	Methylated	DB	quinone oxidoreductase PIG3	2.9E-41	MDP0000303908	800bp 5'UTR	MDC016620.364	C_8592
8	Methylated	DB	VAM6/VPS39-like protein	9.7E-28	MDP0000312750	exon	MDC019094.96	No hit
9	Methylated	DB	AUX/IAA27 B	7.6E-84	MDP0000361838	100 bp 5'UTR	MDC003296.431	C_7332
10	Methylated	DB	U3 small nucleolar ribonucleoprotein protein IMP3	8.3E-57	MDP0000365539	exon and intron	MDC022311.89	C_8153
11	Demethylated	ST	ACT domain-containing protein ACR8-like	9.5E-39	MDP0000162605	exon and intron	MDC000413.350	C_5857
12	Demethylated	ST	dnaJ homolog subfamily A member 3, mitochondrial-like	2.8E-49	MDP0000200636	exon and intron	MDC012610.126	C_24702
13	Demethylated	ST	pentatricopeptide repeat-containing protein	7E-23	MDP0000269242	exon	MDC001244.150	C_14588
14	Demethylated	ST	Embryo defective 2016, putative	4.7E-40	MDP0000299377	exon	MDC011779.270	C_9444
15	Demethylated	ST	TMV resistance protein N-like isoform X1 (NBS-LRR)	1.1E-19	MDP0000378930	exon and intron	MDC000285.554	C_33868
16	Demethylated	ST	Nuclear transcription factor Y subunit B-3-like	8.3E-70	MDP0000818967	150 5'UTR	MDC000337.410	C_34825
17	Methylated	ST	UDP-galactose transporter 2-like	1.5E-52	MDP0000198172	exon and 3'UTR	MDC013793.94	C_33785
18	Methylated	ST	Werner Syndrome-like exonuclease isoform X2	3.2E-96	MDP0000226301	200bp 3'UTR	MDC027549.17	C_9043
19	Methylated	ST	galactose oxidase-like	1.1E-68	MDP0000896660	exon	MDC044052.10	C_30737
20	Demethylated	GT	uncharacterized ATP-dependent helicase C29A10.10c-like	1.3E-46	MDP0000296615	exon and intron	MDC013945.282	C_32594
21	Demethylated	GT	Ribosomal protein kinase G11A-like	1.9E-25	MDP0000153928	intron	MDC019410.118	C_15847
22	Demethylated	GT	acid phosphatase 1-like	1.1E-33	MDP0000186556	exon and 3'UTR	MDC011209.233	C_10934
23	Demethylated	GT	Cation efflux protein/ zinc transporter	6.7E-37	MDP0000230536	exon and intron	MDC003233.264	C_45074
24	Demethylated	GT	Histone acetyltransferase HAC1-like	2.1E-29	MDP0000272113	exon	MDC003556.304	C_21725
25	Demethylated	GT	probable salt tolerance-like protein	1.3E-39	MDP0000551876	exon	MDC015976.77	C_4845
26	Demethylated	GT	Pectinesterase 3	8.7E-22	MDP0000621927	exon	MDC004699.471	C_25510
27	Demethylated	GT	galactose oxidase-like	5.7E-66	MDP0000646516	exon	MDC003162.287	No hit
28	Methylated	GT	serine/threonine-protein phosphatase 7 inactive homolog	8.4E-28	MDP0000158250	intron	MDC000502.364	C_29245
29	Methylated	GT	UDP-galactose/UDP-glucose transporter 3	2E-78	MDP0000165524	exon and intron	MDC000968.94	C_25294
30	Methylated	GT	E3 ubiquitin-protein ligase RING1	3.6E-26	MDP0000189022	exon	MDC011159.221	C_450
31	Methylated	GT	vesicle-associated membrane protein 726 (SNARE protein)	2.2E-8	MDP0000231646	exon	MDC011573.204	C_14669
32	Methylated	GT	Transcriptional corepressor SEUSS isoform X1	8.4E-21	MDP0000278350	exon	MDC007401.275	C_1813
33	Methylated	GT	Cytochrome P450 77A3	2.3E-79	MDP0000322366	exon and intron	MDC017031.336	C_14881
34	Methylated	GT	LRR receptor-like protein 2	1.7E-37	MDP0000600580	exon and intron	MDC040598.17	C_32440
35	Methylated	GT	aldehyde dehydrogenase family 2 member C4-like	9.4E-41	MDP0000662387	380 bp 3'UTR	MDC018307.94	C_63120
36	Methylated	GT	thioredoxin-like protein YLS8 isoform X1	1.1E-27	MDP0000748886	200bp 5'UTR	MDC022326.61	C_7466
37	Methylated	GT	protein Iojap-related, mitochondrial	4.1E-15	MDP0000750612	exon and intron	MDC010033.126	C_10847
38	Methylated	GT	cytochrome P450 71A25-like	1.2E-26	MDP0000843359	exon and down	MDC012381.537	No hit
39	Methylated	GT	IQ-DOMAIN 14-like protein	2.7E-35	MDP0000890508	exon	MDC018371.124	C_27091
40	Demethylated	FS	vam6/Vps39-like protein	1.2E-32	MDP0000134240	exon	MDC003486.520	C_30705
41	Demethylated	FS	NADH-cytochrome b5 reductase 1	1.3E-25	MDP0000164574	exon	MDC002037.232	C_9830
42	Demethylated	FS	DNA mismatch repair protein MSH7-like	2.8E-42	MDP0000306261	exon and intron	MDC017615.201	C_130
43	Demethylated	FS	cation/H(+) antiporter 24-like	2.1E-14	MDP0000310134	exon	MDC020062.119	No hit
44	Demethylated	FS	LRR receptor-like serine/threonine-protein kinase EFR	6.8E-58	MDP0000712707	intron	MDC007491.483	C_32170
45	Methylated	FS	metal tolerance protein 10-like	2.3E-57	MDP0000130310	200bp 5'UTR	MDC018194.83	No hit
46	Methylated	FS	cysteine proteinase	2.2E-9	MDP0000189200	exon and 3'UTR	MDC009544.135	C_2542
47	Methylated	FS	ATP-dependent Clp protease ATP-binding subunit clpX-like	1.8E-8	MDP0000719271	150bp 5'UTR	MDC014085.237	C_15418

### Global expression analysis of MSAP fragments associated genes

**Fig 3:**
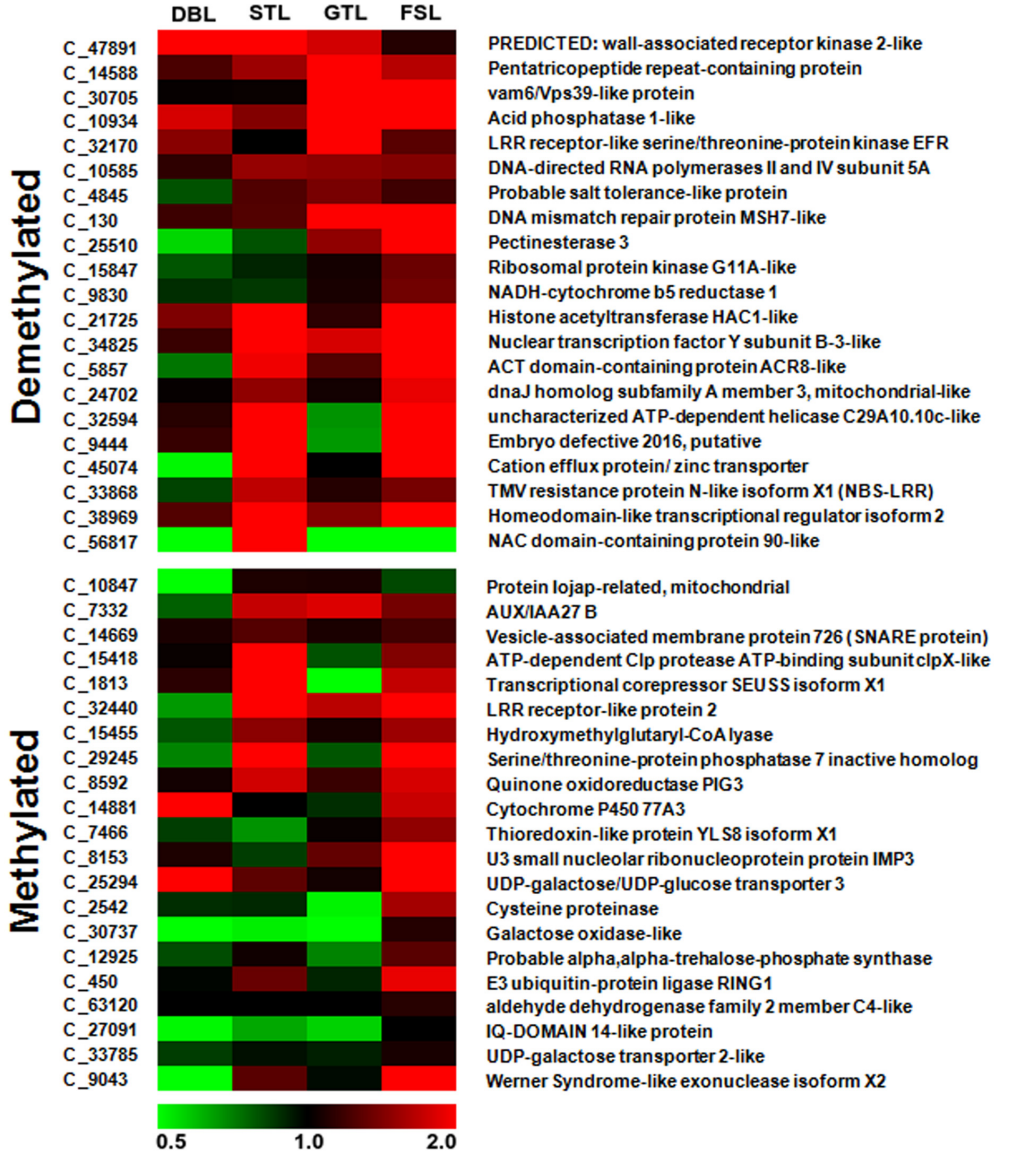
Heat map representation of relative expression based on FPKM values of differentially methylated fragments in various developmental tissues as identified in MSAP analysis. The majority of demethylated fragments showed upregulation under low chill conditions as compared to high chill conditions. The relative expression of majority of methylated fragments was downregulated. The relative FPKM values were used to generate the heat-map using MeV4. The color scale at the bottom represents the expression level, where red, green and black colors indicate upregulation, downregulation and unaltered expression, respectively. Contig number (starting with C_) and their annotation are given on the left and right side of the heat map, respectively.

In order to determine the effect of methylation change on the expression of MSAP fragments associated genes in various developmental stages during dormancy and fruit set, RNA-seq analysis of eight samples representing 2 dormant stages of floral spur bud (DB and ST), one stage of actively growing floral bud (GT) and one stage of fruit initiation (FS) under high and low chill conditions was performed (Table 1). The paired-end sequencing generated 97,960,812 raw reads of which 7,080,502 low quality reads were filtered out. The *de novo* assembly of 90,880,310 high quality reads resulted in 68,455 contigs with N50 of 766 bp. Out of 47 randomly selected MSAP fragments, expression of 42 was found in all the analyzed tissues, while remaining 5 genes exhibit either very low or no expression in any of the tissues. From the 42 MSAP fragments, the expression of majority of demethylated fragments associated genes was observed to exhibit high expression while majority of methylated contigs were either down-regulated or moderately expressed (Fig 3), which suggest that expression of these genes is affected by their DNA methylation. Overall, this analysis shows the moderate correlation between methylation status and level of gene expression.

**Fig 4:**
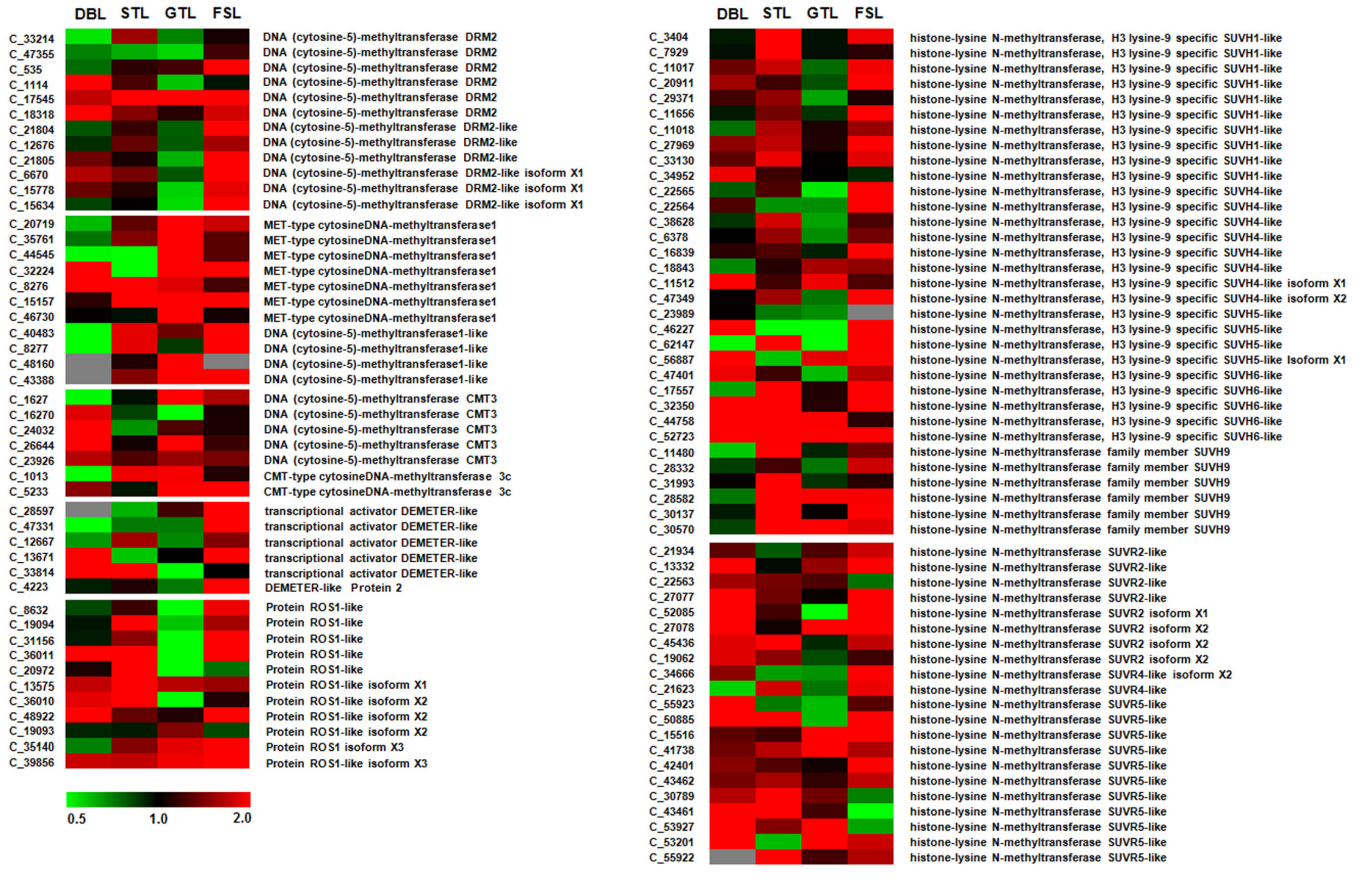
Heat map representation of relative expression based on FPKM values of DNA methyltransferases, DNA glycosylases and histone methyltransferases in various developmental tissues. The relative FPKM values were used to generate the heat-map using MeV4. The color scale at the bottom represents the expression level, where red, green and black colors indicate upregulation, downregulation and unaltered expression, respectively. Contig number (starting with C_) and their annotation are given on the left and right side of the heat map, respectively.

The expression of genes involved in DNA and histone methylation/demethylation was also analyzed in various developmental stages under differential chilling conditions (Fig 4). In our RNA-seq data, twelve transcripts were annotated as *DOMAINS REARRANGED METHYLTRANSFERASE 2* (*DRM2*) and *DRM2*-like genes, which are involved in *de novo* DNA methylation. In DBL-vs-DBH comparison, only four transcripts (C_6670, C_1114, C_17545 and C_18318) of *DRM2* and *DRM2*-like were found to be upregulated (>1.5 fold change). However, in FSL-vs-FSH comparison, expression of nine *DRM2* and *DRM2*-like transcripts was found to be upregulated, while expression of remaining three transcripts (C_1114, C_33214 and C_47355) was found to be unaltered. Although, most of the *DRM2* and *DRM2*-like transcripts (six out of twelve) were found to exhibit downregulation (<0.75 fold change) in GTL-vs-GTH, the expression of all the transcripts (except, C_8277 and C_40483 with un-altered expression) of *DNA METHYLTRANSFERASE1 (MET1*) and *MET1*-like transcripts, which maintain CG methylation, was found to be upregulated in GTL-vs-GTH comparative condition (Fig 4). In addition, six out of eleven *MET1* and *MET1*-like transcripts were also found to be upregulated in STL-vs-STH (C_35761, C_8276, C_15157, C_40483, C_8277 and C_43388) and FSL-vs-FSH (C_20719, C_32224, C_15157, C_40483, C_8277 and C_43388) comparisons. Similarly, out of seven transcripts of *CHROMOMETHYLASE 3* (*CMT3*), which maintain CHG methylation, five were found to be upregulated in DBL-vs-DBH (C_5233, C_16270, C_23926, C_24032 and C_26644) and GTL-vs-GTH (C_1013, C_1627, C_5233, C_23926 and C_26644) comparisons (Fig 4). In addition, expression of DNA glycosylases, *DEMETER* (*DME*) and *REPRESSOR OF SILENCING1* (*ROS1*), which are involved in active demethylation of cytosine, was also analyzed. The expression of five out of six *DME*-like transcripts was upregulated in FSL-vs-FSH, while only two exhibited upregulation in DBL-vs-DBH (C_13671 and C_33814) and STL-vs-STH (C_12667 and C_33814). Whereas, in GTL-vs-GTH comparison, most of the *DME*-like transcripts were either downregulated or have unaltered expression. The high relative expression of majority of *ROS1*-like transcripts was observed to exhibit upregulation in various comparative conditions. Out of eleven *ROS1*-like transcripts, expression of five, eight, four and eight transcripts was found to be upregulated DBL-vs-DBH, STL-vs-STH, GTL-vs-GTH and FSL-vs-FSH comparisons, respectively (Fig 4). These results suggest that, the *de novo* DNA methylation by *DRM2* during dormant bud (DB) and fruit set (FS) stages and its maintenance by *MET1* and *CMT3* during bud break (GT) and fruit set with concomitant downregulation of *DME*-like and *ROS1*-like transcripts during bud break might be responsible for high percentage of cytosine methylation under low chill conditions even after dormancy release (Table 2).

Since, histone methylation marks are observed to be linked with DNA methylation, expression of histone lysine methyltransferase (HMTase), including *SUVH-like* and *SUVR-like* transcripts was also analyzed. In total, 33 transcripts were found to be annotated as *SUVH-like*, which belong to different classes including, *SUVH1*-like (10 transcripts), *SUVH4*-like (8 transcripts), *SUVH5*-like (4 transcripts), *SUVH6*-like (5 transcripts) and *SUVH9* (6 transcripts). The relative expression of seven out of ten *SUVH1*-like transcripts was found to be upregulated in each STL-vs-STH and FSL-vs-FSH comparisons. Similarly, majority of *SUVH4*-like (five out of eight), *SUVH5*-like (three out of four) and *SUVH9* (five out of six) transcripts were found to be upregulated in FSL-vs-FSH. In addition, five transcripts of *SUVH9* were also found to be upregulated in STL-vs-STH, while majority of *SUVH6*-like transcripts were found to be upregulated in all the comparative conditions, except in GTL-vs-GTH, where only two *SUVH6*-like transcripts were upregulated. Similarly, majority of *SUVR2*-like transcripts were also found to be upregulated in DBL-VS-DBH and FSL-VS-FSH comparative conditions, while majority of *SUVR5*-like transcripts were found to be upregulated in all the comparative conditions, except GTL-VS-GTH. Only two transcripts were annotated as *SUVR4*-like, both of them exhibit upregulation in FSL-VS-FSH comparisons (Fig 4).

### Quantitative-real time PCR analysis of genes associated with methylation changes

**Fig 5:**
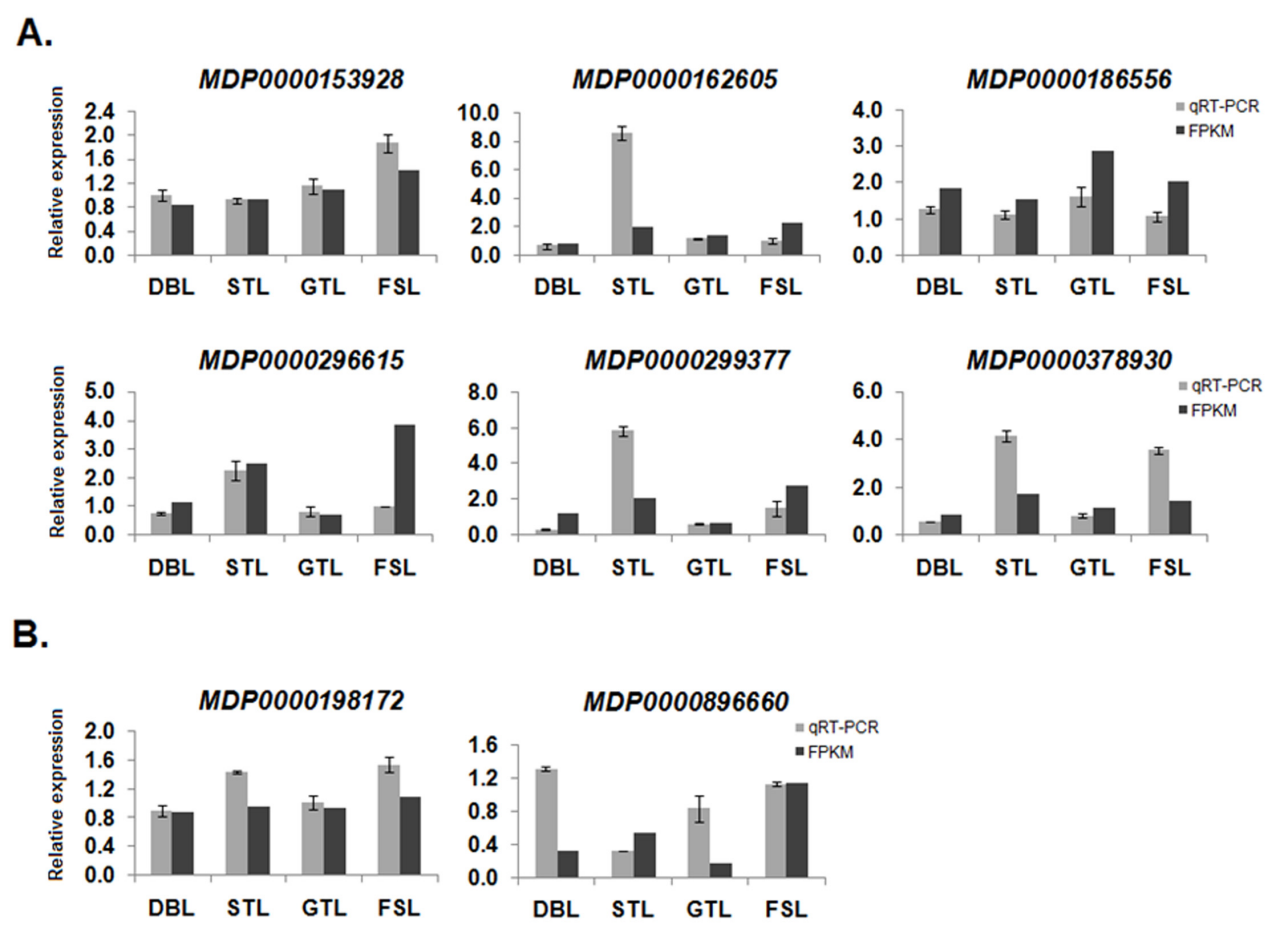
**Relative expression of randomly selected (A) demethylated and (B) methylated genes analyzed through qRT-PCR.** Pearson correlation coefficient between fold change of FPKM based expression and qRT-PCR was 0.431 (p-value<0.0005).

The FPKM based expression obtained through RNA-Seq for genes associated with MSAP loci was validated using qRT-PCR (Fig 5). In total, eight genes, which have methylation change either within the gene body (intron/exon) or upstream/downstream regions were selected for qRT-PCR analysis. Out of these, six genes *viz*. MDP0000153928, MDP0000162605, MDP0000186556, MDP0000296615, MDP0000299377 and MDP0000378980 were demethylated, while, two genes viz. MDP0000198172, and MDP0000896660 were methylated in different developmental stages (Table 4). The FPKM based expression values were found to be in accordance with qRT-PCR expression values. Among six demethylated genes, expression of four (MDP0000162605, MDP0000186556, MDP0000299377 and MDP0000378930) was found to be up-regulated (>1.5 fold change) in samples of low chill conditions where demethylated fragments of these genes were observed in MSAP analysis (Fig 5). While the expression of other two demethylated genes, MDP0000153928 and MDP0000162605, was observed to be unaltered in corresponding samples. Similarly, from the two methylated genes, only one (MDP0000896660) was found to be downregulated in corresponding samples under low chill conditions as compared to high chill conditions, while the expression of MDP0000198172 was either up-regulated or remained unaltered (Fig 5). From the qRT-PCR analysis, it was observed that MSAP methylation status of genes was highly correlated with their expression induction or inhibition. For instance, four out of six demethylated genes (MDP0000162605, MDP0000186556, MDP0000299377 and MDP0000378930), were upregulated in samples where demethylation of these genes were observed in MSAP analysis. Similarly, the correlation between methylation and suppression of expression was observed for methylated gene, MDP0000896660, which showed downregulated expression in corresponding sample where methylated fragment of this gene was observed.

### Bisulfite sequencing of polymorphic fragments

In order to accurately determine the level of cytosine methylation of polymorphic MSAP loci and to correlate with their expression levels, four loci; MDC013945.282 (MDP0000296615), MDC019410.118 (MDP0000153928), MDC011209.233 (MDP0000186556) and MDC044052.10 (MDP0000896660) were randomly selected for bisulfite sequencing. The methylation pattern of three types of cytosine sequence contexts CG, CHH and CHG (where H represents the A, T or C), were analyzed. In all the four bisulfite sequenced loci, maximum methylated cytosines were found to be of CG type (15), followed by CHH (13) and CHG (5) (S1, S2, S3 and S4 Figs). Interestingly, under differential chilling conditions, the total methylation events were found to be higher under low chill conditions with 20 methylated cytosines, while under high chill conditions, only 13 methylated cytosines were observed in any of the sequence context. Irrespective of chilling conditions, the number of methylated cytosines during the dormant period (DBL, DBH, STL and STH) was relatively higher as compared to the active growth period (GTL, GTH, FSL and FSH). However, among dormant stages, thirteen methylated cytosines were identified under low chill conditions (DBL and STL) as compared to only seven methylated cytosines under high chill conditions (DBH and STH). However, during the active growth period (GT and FS), the number of methylated cytosine were comparable under both the chilling conditions. The methylation status of all the bisulfite sequenced loci, except MDP0000896660, indicates that low chill conditions have comparatively high cytosine methylation even after the dormancy release, which is in agreement with the MSAP scoring. The MDP0000296615 gene (MDC013945.282 loci), was found to have seven methylated cytosines in different sequence contexts, where most of them were present in DBL, STL, and FSL, while, methylated cytosines were found at positions 61 in STH and 233 in DBH, GTH and FSH (S1 Fig). Moreover, the percentage of methylated cytosines was observed to be high under low chill conditions with 8.57% in each DBL and STL and 5.71% in FSL, except in GTL, where it was only 2.85%. Whereas, constantly low (2.85%) percentage of methylated cytosines was observed in all the stages under high chill conditions. Therefore, it appeared that the gene body of MDP0000296615 is hyper-methylated under low chill conditions as compared to high chill conditions. In case of MDP0000153928 gene (MDC019410.118 loci), methylated cytosines were observed at position 6 in DBL and GTL, and at position 29 in STL with constant percentage of methylated cytosines (5%) in these samples, while no methylated cytosine was observed in FSL, DBH, STH, GTH and FSH (S2 Fig). This observation indicates that MDP0000153928 gene body was also hyper-methylated under low chill conditions. The *MDP0000186556* gene (MDC011209.233 loci), was found to have methylated cytosines at positions 52 and 58 in DBL and at position 81 and 159 in STL and GTL, respectively. While, the cytosines at positions 126 and 131 in DBH, and at position 128 and 159 in STH were observed to be methylated (S3 Fig). The percentage of methylated cytosines under high chill conditions was observed to be constant with 3.7% in both DBH and STH, thereafter, no methylated cytosine was observed during active growth phase. While, under low chill conditions, the percentage of methylated cytosine was found to be 3.7% during DBL, thereafter, decreased towards the dormancy release, with 1.88% methylated cytosine in STL and GTL. In case of *MDP0000896660* gene (MDC044052.10 loci), under low chill conditions, the methylated cytosines were observed at position 88 and 272 in STL and at position 164 and 272 in GTL and FSL, respectively. While under high chill conditions, the nucleotide positions at 150 in DBH, 78 in GTH and nucleotide positions 45, 200 and 272 in FSH were found to have methylated cytosines (S4 Fig). Although, similar to other bisulfite sequenced genes under low chill conditions, gene body of *MDP0000896660* was found to be hyper-methylated during dormant period in STL with 3.27% methylated cytosines, thereafter decreased towards active growth (GTL and FSL) with 1.63% methylated cytosine, increases in cytosine methylation under high chill conditions was observed during fruit set (FSH) with 4.91% of methylated cytosines. From the qRT-PCR analysis and bisulfite sequencing of these four MSAP fragments, it is observed that methylation level and gene expression is moderately correlated.

## Discussion

In temperate fruit crops, the low temperature during winter dormancy period is crucial for the normal phenological traits and quality and yield of the crop. Most of the previous studies related to dormancy release in various plants species have been based on the gene expression using global transcriptome analysis [40–43]. However, the regulatory network behind chilling requirement in relation to fruit development is highly complex. The epigenetic processes, like DNA methylation/demethylation, have been shown to play important roles in regulating gene expression in response to diverse environmental signals [21, 44]. Apple, being a temperate fruit crop, also requires adequate chilling temperature during dormancy period to resume normal active growth in response to warm temperature. While, lack of sufficient chilling leads to irregular bud break and delayed flowering, which consequently have detrimental effects on yield and quality of fruits. Therefore, in order to study the consequences of differential chilling availability on epigenetic regulation during winter dormancy, MSAP and RNA-Seq approaches have been employed.

In the present study, the temporal changes in methylation status of 5'-CCGG-3' tetra-nucleotide in apple genome were analyzed in four developmental stages from dormant bud to fruit set (DB, ST, GT and FS) under differential availability of chilling using MSAP technique. The MSAP profiles reveal the significant changes in methylation status during different temporal development stages of apple bud and fruit set under differential chilling availability (Table 2, Table 3). The results indicate the biological importance of epigenetic modifications in response to chilling during dormancy release and subsequent fruit set in apple. The methylation patterns of comparable stages, showed decrease in demethylation events under low chill condition as compared to high chill conditions, which suggests that re-initiation of active growth (bud break) and fruit set is linked with progressive demethylation events under ideal high chill conditions. In *Arabidopsis*, early flowering has been found to be associated with reduced 5-methyl cytosine level in vernalized (prolonged cold treatment) and 5-azacytidine (a DNA demethylating agent) treated plants (Burn et al. 1983). The decrease in DNA methylation at 5'-CCGG-3' sites with dormancy release was also reported in potato tubers and chestnut [45–46]. The previous study in pepper reported increase in demethylation during seed germination [33]. The low temperature associated demethylation in subsequent reproductive growth was also reported in wheat, rapeseed and *Arabidopsis* [11, 27–30]. Thus, it is evident from the previous studies, that the acquisition of low temperature has been associated with decrease in methylation and/or increase in demethylation during active growth phase of the plant. Interestingly, in the present study, availability of less chilling during dormancy period was found to be associated with less demethylation of 5'-CCGG-3' sequences in apple genome. Moreover, the methylation was also observed to be progressively decreased along the chilling mediated dormancy release and subsequent fruit set in apple (Table 2). The similar developmental stage specific changes in drought stress induced DNA methylation were also observed in rice [47].

The sequencing and gene ontology (GO) annotation of 47 MSAP fragments reveal that, the genes which undergo methylation changes are involved in diverse physiological and regulatory pathways (Table 4). These genes are broadly related to GO terms, such as response to stimulus, transcriptional regulation, catalytic activity, regulation of biological processes, cellular and metabolic processes, etc. The genes encoding pentatricopeptide repeat protein (PPR) and thioredoxin-like were previously reported to be involved in transcriptional regulation of gene expression in plants cell organelles [48–50]. In our analysis, the *PPR* gene was found to be demethylated in silver tip (ST) stage, where plant is committed to resume active growth. While, the *thioredoxin*-like gene was found to be methylated in green tip (GT) stage. The gene annotated as transcriptional corepressor SEUSS isoform X1 (SEU), was found to be methylated in GT stage, where floral meristem development is initiated. The SEU along with LEUNIG (LUG) was previously reported to be involved in repression of *AGAMOUS* (*AG*), a C class floral homeotic MADS gene [51]. Therefore, our results suggest that transcriptional SEU might be epigenetically regulated through cytosine methylation to remove repressive effect of SEU-LUG repressor complex on *AG* expression to initiate the flower development in apple. In *Arabidopsis*, *HAC1* mutants exhibited late-flowering phenotype and therefore implicated to be involved in flowering time regulation [52]. The increase in histone acetyltransferase activity was previous reported in germinating *Zea mays* embryos [53]. Interestingly in our study, the histone acetyltransferase *HAC1*-like gene was found to be demethylated in GT stage, where plant resumes its active growth after dormant period. Moreover, a homeodomain containing *Arabidopsis* genes, *FWA* and *BEL1* was reported to be involved in reproductive tissue development, where expression of *FWA* was epigenetically regulated through hypo-methylation of its promoter and coding region [54, 55]. In our analysis, *homeodomain*-like gene was also found to be demethylated at DB stage, however the gene identified in present analysis was similar to Arabidopsis homeodomain-like transcription regulator which was found to be expressed mainly during the reproductive organ development [56]. The auxin responsive gene has also been found to have altered expression during dormancy release in plants [3, 57]. In present analysis, the gene *AUX/IAA27 B*, of AUX/IAA family, was found to be methylated. Gene encoding NAC domain containing protein was found to be demethylated during the chilling acquisition that might have some role in flowering regulation. Previously *LOV1*, a NAC transcription factor was also found to be involved in flowering time regulation by negatively regulating the expression of *CONSTANS* (*CO*) and enhancing cold tolerance in transgenic Arabidopsis [58]. Recently, Arabidopsis *NAC050* and *NAC052* have been shown to regulate flowering time by associating with histone demethylase, JMJ14 [59].

In addition, the genes involved in RNA/DNA processing and transcriptional regulation, including RNA polymerase, U3 small nucleolar ribonucleoprotein, exonuclease, nuclear transcription factor Y, IQ-DOMAIN 14-like, DNA mismatch repair protein MSH7-like and E3 ubiquitin-protein ligase RING1 were also found to be differentially methylated in present analysis. Similarly, the gene involved in DNA/RNA processing and transcriptional regulation has been found to be differentially methylated in ryegrass [60] and maize [61]. Previous studies in poplar, kiwifruit and raspberry have reported the differential expression of stress related genes during the dormancy release [62–64]. In the present analysis, the genes involved in stress response, including CYP71A25-like, cation efflux protein, salt tolerance-like protein and quinone oxidoreductase PIG3 homolog were also found to be either methylated or demethylated, and exhibit differential expression (Table 4). In addition, wall-associated receptor kinase2-like, pectinesterase3, NADH-cytochrome b5 reductase1, aldehyde dehydrogenase family2 member C4-like, cysteine proteinase and SNARE protein involved in cellular and metabolic processes were also found to have altered methylation patterns (Table 4).

In *Arabidopsis*, the transcriptional regulation of genes was found to be determined by methylation levels within their gene bodies. Interestingly, these genes were found to be functionally more important as compared to the genes which are lacking methylation sites within their gene bodies [65, 66]. In addition, gene body methylation was also reported in other plants, like rice and sorghum [34, 47, 67]. The methylation changes at 5' and 3' regions of gene bodies and their flanking regions, was found to have regulatory effect on gene expression [68]. Similarly, in the present analysis, all the MSAP fragments reveal methylation in either gene body or 5' and 3' flanking regions (Table 4), which suggest that methylation may provide an additional regulatory mechanism to alter the transcription of these genes. In qRT-PCR analysis, expression of five out of eight genes associated with the methylated and demethylated fragments, was observed in accordance with their DNA methylation status (Fig 5). Therefore, our results suggest that changes in methylation status within the gene bodies are correlated with their gene expression patterns. These genes are involved in different biological processes and molecular functions. For instance, gene encoding UDP-galactose transporter (MDP0000198172) is involved in transport of UDP-galactose in golgi lumen for galactosylation of lipids, proteins and cell wall matrix polysaccharide [69, 70]. The embryo defective gene (MDP0000299377), that might have some role in embryo development [71], was found to be demethylated at ST stage, where plant was committed to grow in response to warm temperature. Similarly, the gene encoding ACT domain containing protein, previously reported to act as metabolism regulatory or sensory proteins in plants [72], was also found to be demethylated in ST stage. Moreover, the MSAP analysis represents only the 5'-CCGG-3' methylation, the cytosine methylation changes at other sequence contexts may also regulate the gene expression. Therefore, to accurately correlate the gene expression with gene body methylation percentage, bisulfite sequencing of randomly selected four MSAP fragments was performed. The ATP-dependent helicase belonging to DNA2/NAM7 helicase family (MDP0000296615), involved in replication/transcription, was found to be hyper-methylated, however, its expression pattern was moderately correlated with methylation level (Fig 5, S1 Fig). In contrast, high correlation between methylation level and expression pattern was observed for hyper-methylated MDP0000153928 gene (*ribosomal protein kinase* G11A-like) involved in signal transduction and metabolic regulation (Fig 5, S2 Fig). Similarly, the *acid phosphatase 1*-like gene (MDP0000186556), involved in phosphate energy metabolism [73], has low methylation level under low chill condition, which correlates with its expression pattern (Fig 5, S3 Fig). The hyper-methylation of *galactose oxidase*-like gene (MDP0000896660) under high chill condition was also found to be correlated with its expression pattern (Fig 5, S4 Fig). The *galactose oxidase* gene is involved in the production of reactive oxygen species, particularly H_2_O_2_ that also acts as signaling molecule [74]. Moreover, bisulfite sequencing of these genes suggests that low chilling might be associated with hyper-methylation of gene body that might result into suppression of their expression. Therefore, these results indicate that gene body methylation is a determining factor in regulating the gene expression. Moreover, FPKM based expression profiles of other genes associated with MSAP fragments also suggest that methylation status of genes was also correlated with their transcriptional activation or inhibition (Fig 3). The similar association between DNA methylation and transcriptional regulation of genes has also been reported in *Arabidopsis* [65].

In addition, the expression analysis of DNA methyltransferases, DNA gylcosylases and histone methyltransferases showed that chilling availability during winter dormancy has profound impact on the expression of genes encoding enzymes which actively catalyze the DNA methylation/demethylation and histone methylation. For instance, under low chill conditions, high percentage of fully methylated MSAP fragments was in agreement with high expression of *MET1* and *CMT3* methyltransferases in addition to low expression of *DME*-like and *ROS1*-like DNA glycosylases during active growth (GTL). Whereas, under high chill conditions, the gradual decrease in methylated fragments along the dormancy release and fruit set, was consistent with down-regulated expression of *MET1* and *CMT3* transcripts and high expression of DNA glycosylases. It has been previously reported that, some of the histone methylation marks are correlated with DNA cytosine methylation, which suggests cross talk between DNA methylation and histone methylation marks [75–78]. The H3K9, histone methylation mark was reported to be associated with DNA cytosine methylation at CHG sequence context [79], while, H3K4me1 mark has been reported to be associated with DNA methylation at CG sequence context [80]. In MSAP, based on methylation-sensitivity of the restriction enzymes (*Msp*I/*Hpa*II), it can be assumed that CHG (^5m^CCGG) and CG (C^5m^CGG) sequence contexts are mainly represented by hemi-methylated and fully methylated fragments, respectively. However, this assumption is inexact, since the different cytosine methylation combinations at 5'-CCGG-3' restriction site cannot be detected by activity of *Msp*I/*Hpa*II [22]. In the present analysis, the high percentage of hemi-methylated fragments in STL and FSL was in correlation with concomitant upregulation of *SUVH4*, *SUVH6* and *SUVH9* transcripts, involved in methylation of H3K9 mark [79,81–83], which further reinforce the presence of higher CHG methylation during STL and FSL, under low chill condition. In addition, *SUVR4* and *SUVR5*, which are also involved in methylation of H3K9 histone mark [84, 85], were observed to be upregulated under low chill conditions in most of the comparative conditions. Conclusively, these findings suggest that the availability of chilling modulates the complex interplay between active and passive demethylation (replication and base excision), and associated *de novo* methylation and its maintenance by altering the gene expression of participating enzymes which are responsible for methylation changes.

## Conclusion

To best of our knowledge, this is the first report of changes in methylation patterns during dormancy release and fruit set in apple, under differential chilling availability. The analysis of developmental stage specific changes in methylation level suggested that adequate (high) chilling availability during dormancy period is associated with reduced methylation of apple genome along the dormancy release and fruit set. Moreover, the chilling availability appears to modulate the expression of genes involved in active DNA methylation and demethylation. In addition, the genes encoding proteins involved in various biological processes like transcriptional regulation, catalysis, transport, defense, response to stimulus etc. were found to have altered methylation status under differential chilling. Thus, our results demonstrate that epigenetic modification through cytosine methylation is an important regulatory mechanism to control the dormancy release in apple and is likely to be modulated by chilling acquisition during dormancy period.

## Supporting Information

S1 Fig Bisulfite sequencing analysis of MDC013945.282 MSAP fragment in eight different samples. Sequence of top strand (ORG) represents the non-bisulfite converted locus and was cloned from the genomic DNA of bud samples.
(DOCX)

S2 Fig Bisulfite sequencing analysis of MDC019410.118 MSAP fragment in eight different samples. Sequence of top strand (ORG) represents the non-bisulfite converted locus and was cloned from the genomic DNA of bud samples.
(DOCX)

S3 Fig Bisulfite sequencing analysis of MDC011209.233 MSAP fragment in eight different samples. Sequence of top strand (ORG) represents the non-bisulfite converted locus and was cloned from the genomic DNA of bud samples.
(DOCX)

S4 Fig Bisulfite sequencing analysis of MDC044052.10 MSAP fragment in eight different samples. Sequence of top strand (ORG) represents the non-bisulfite converted locus and was cloned from the genomic DNA of bud samples.
(DOCX)

S1 Table List of adaptors and primers used for MSAP assay. (DOCX)

S2 Table List of primers used to amplify bisulfite-converted DNA. (DOCX)

S3 Table List of primers used in qRT-PCR analysis. (DOCX)

S4 Table Chi-square test statistic for testing independence between methylation level and chilling conditions as well as during developmental stages. (DOCX)
