# Supplementary material for: Chilling-Mediated DNA Methylation Changes during Dormancy and Its Release Reveal the Importance of Epigenetic Regulation during Winter Dormancy in Apple (Malus x domestica Borkh.)
Source: PLoS One. 2016 Feb 22;11(2):e0149934. doi: 10.1371/journal.pone.0149934 (PMC4763039; doi:10.1371/journal.pone.0149934)
Supplement: S2 Fig — Sequence of top strand (ORG) represents the non-bisulfite converted locus and was cloned from the genomic DNA of bud samples. (DOCX) [file pone.0149934.s002.docx]

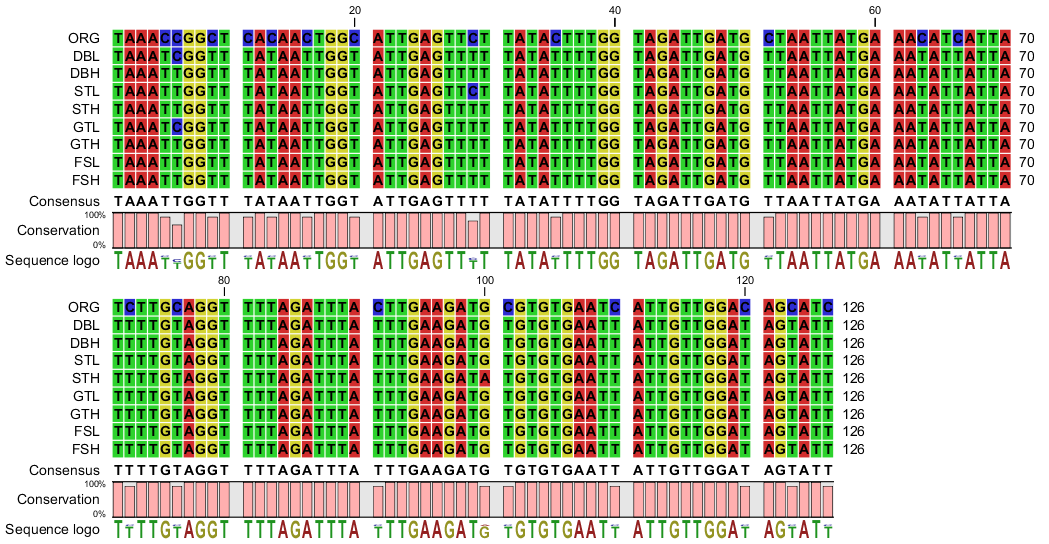


**S2 Fig.** Bisulfite sequencing analysis of MDC019410.118 MSAP fragment in eight different samples. Sequence of top strand (ORG) represents the non-bisulfite converted locus and was cloned from the genomic DNA of bud samples.
