# Supplementary material for: Chilling-Mediated DNA Methylation Changes during Dormancy and Its Release Reveal the Importance of Epigenetic Regulation during Winter Dormancy in Apple (Malus x domestica Borkh.)
Source: PLoS One. 2016 Feb 22;11(2):e0149934. doi: 10.1371/journal.pone.0149934 (PMC4763039; doi:10.1371/journal.pone.0149934)
Supplement: S1 Table — (DOCX) [file pone.0149934.s005.docx]

**S1 Table.** List of adaptors and primers used for MSAP assay.

| **Primers/adapters** | **Sequence (5' to 3' )** |
| --- | --- |
| *Eco*RI adapter | 5'-CTCGTAGACTGCGTACC-3' |
|  | 5'-AATTGGTACGCAGTCTAC-3' |
| *Hpa*II/*Msp*I adapter | 5'-GATCATGAGTCCTGCT-3' |
|  | 5'-CGAGCAGGACTCATGA-3' |
| *Eco*RI pre-selective primer | 5'-GACTGCGTACCAATTA-3' |
| *Hpa*II/*Msp*I pre-selective primer | 5'-ATCATGAGTCCTGCTCGT-3' |
| *Eco*RI selective primers |  |
| E1 | 5'-GACTGCGTACCAATTA+AC |
| E2 | 5'-GACTGCGTACCAATTA+CG |
| E3 | 5'-GACTGCGTACCAATTA+CT |
| E4 | 5'-GACTGCGTACCAATTA+GT |
| *Hpa*II/*Msp*I selective primer |  |
| HM1 | 5'-ATCATGAGTCCTGCTCGT+TG |
| HM2 | 5'-ATCATGAGTCCTGCTCGT+AG |
| HM3 | 5'-ATCATGAGTCCTGCTCGT+AC |
| HM4 | 5'-ATCATGAGTCCTGCTCGT+TT |
