# Supplementary material for: Chilling-Mediated DNA Methylation Changes during Dormancy and Its Release Reveal the Importance of Epigenetic Regulation during Winter Dormancy in Apple (Malus x domestica Borkh.)
Source: PLoS One. 2016 Feb 22;11(2):e0149934. doi: 10.1371/journal.pone.0149934 (PMC4763039; doi:10.1371/journal.pone.0149934)
Supplement: S2 Table — (DOCX) [file pone.0149934.s006.docx]

**S2 Table.** List of primers used to amplify bisulfite-converted DNA

| **Sl. No.** | **MDC ID** | **MDP ID** | **Sequence (5' to 3')** |
| --- | --- | --- | --- |
| 1 | MDC013945.282 | MDP0000296615 | Fwd- AAATGTTAATGAAGTGAGTTATAATAAG |
|  |  |  | Rev- AAATATTTCRAACAACATATATATTCC |
| 2 | MDC019410.118 | MDP0000153928 | Fwd- TAAATYGGTTTATAATTGGTATTGAG |
|  |  |  | Rev- AATACTATCCAACAATAATTCACAC |
| 3 | MDC011209.233 | MDP0000186556 | Fwd- AGGTAATGTAATATATTGGGTTTGG |
|  |  |  | Rev- TCCTTTTCCTCTTTCCTTCTATTTCTTC |
| 4 | MDC044052.10 | MDP0000896660 | Fwd- TTTTTAATAGAATTGAGGTTAGAGGTATTTAG |
|  |  |  | Rev- TTCATACRTTATTAACCCTACCTAATATATATTC |
