# Supplementary material for: Chilling-Mediated DNA Methylation Changes during Dormancy and Its Release Reveal the Importance of Epigenetic Regulation during Winter Dormancy in Apple (Malus x domestica Borkh.)
Source: PLoS One. 2016 Feb 22;11(2):e0149934. doi: 10.1371/journal.pone.0149934 (PMC4763039; doi:10.1371/journal.pone.0149934)
Supplement: S3 Table — (DOCX) [file pone.0149934.s007.docx]

**S3 Table.** List of primers used in qRT-PCR analysis

| **Sl. No.** | **MDP ID** | **Sequence (5' to 3')** |
| --- | --- | --- |
| 1 | MDP0000296615 | Fwd- GAAGACAGTCGGCATCTCACTCTT |
|  |  | Rev- AACCAACTTCACGAGTCGATCTG |
| 2 | MDP0000153928 | Fwd- CTCCCTAAAACCCCCATTTCTT |
|  |  | Rev- CCGGGTTTGAGAGAAATTGAAG |
| 3 | MDP0000186556 | Fwd- CATCCCAATCCGTCATCCA |
|  |  | Rev- AATCTCCAGCTGTCGCAGTAGAG |
| 4 | MDP0000299377 | Fwd- AGTGAGGCCAGAACAAAGGTTATG |
|  |  | Rev- CCCGTTGTCTTCATGTGATTCA |
| 5 | MDP0000378930 | Fwd- GGACCCCGGACTTTACAGTTACT |
|  |  | Rev- CAGCTATGGATGGGTGGATCAT |
| 6 | MDP0000198172 | Fwd- ATTGTCCTGTCTTGCACCATTG |
|  |  | Rev- AAGGACTTGAAATGACACAGCAGTA |
| 7 | MDP0000896660 | Fwd- GGCCAACAACCTATCAAGTTCAAG |
|  |  | Rev- GCTGGTGAACCACGTAGAGAATG |
| 8 | MDP0000162605 | Fwd- GGTGTGCCCACGATATCATAATAA |
|  |  | Rev- ATGTCCACGTCGGATAAAGTAGGT |
