# Supplementary material for: Chilling-Mediated DNA Methylation Changes during Dormancy and Its Release Reveal the Importance of Epigenetic Regulation during Winter Dormancy in Apple (Malus x domestica Borkh.)
Source: PLoS One. 2016 Feb 22;11(2):e0149934. doi: 10.1371/journal.pone.0149934 (PMC4763039; doi:10.1371/journal.pone.0149934)
Supplement: S4 Table — (DOCX) [file pone.0149934.s008.docx]

**S4 Table.** Chi-square test statistic for testing independence between methylation level and chilling conditions as well as during developmental stages.

|  | **DBL-vs-DBH** | **STL-vs-STH** | | **GTL-vs-GTH** | | **FSL-vs-FSH** |
| --- | --- | --- | --- | --- | --- | --- |
| No change | 561 | 543 | | 538 | | 507 |
| Demethylation events | 39 | 34 | | 27 | | 15 |
| Methylation events | 2 | 8 | | 14 | | 18 |
|  |  |  | |  | |  |
| **Chi-square test statistics** | | | | | | |
|  | DBL-vs-DbH and STL-vs-STH | | DBL-vs-DBH and GTL-vs-GTH | | DBL-vs-DBH and FSL-vs-FSH | |
| χ2 | 3.993 | | 11.219 | | 22.898 | |
| degree of freedom | 2 | | 2 | | 2 | |
| P-value | >0.05 | | <0.05 | | <0.05 | |
| Association between temperature and methylation | No significant association | | Significant association | | Significant association | |
